# Student and Faculty Diversity in Medical School Selection

**DOI:** 10.1001/jamanetworkopen.2025.33727

**Published:** 2025-10-21

**Authors:** Mytien Nguyen, Sarwat I. Chaudhry, Alexandra M. Hajduk, Gbenga Ogedegbe, David Henderson, Shruthi Venkataraman, Dowin Boatright

**Affiliations:** 1 Department of Immunobiology, Yale University School of Medicine, New Haven, Connecticut; 2 Section of General Internal Medicine, Department of Medicine, Yale School of Medicine, New Haven, Connecticut; 3 Section of Geriatrics, Department of Internal Medicine, Yale School of Medicine, New Haven, Connecticut; 4 Institute for Excellence in Health Equity, New York University Grossman School of Medicine, New York; 5 Department of Family Medicine, University of Connecticut School of Medicine, Farmington; 6 Department of Emergency Medicine, New York University Langone Health, New York, New York

## Abstract

This cohort study describes US matriculants’ perceptions of the importance of student and faculty diversity in selecting a medical school.

## Introduction

Fostering diverse student bodies and faculties in medical schools enriches learning environments, enhances cultural competence, and ultimately improves patient care in the US’s increasingly multicultural society.^1,2,3,4^ Nevertheless, a 2025 study found a decline in American Indian, Alaska Native, Native Hawaiian, or Pacific Islander; Black; and Hispanic medical school matriculants.^5^ Understanding how matriculants value student and faculty diversity can provide insight into the evolving priorities of the next generation of physicians. This study investigates trends over the past decade in how medical students have prioritized student and faculty diversity when selecting a medical school.

## Methods

This cohort study used deidentified data and is exempt from review and informed consent per criteria from the New York University Langone Health institutional review board. This study is reported following the Strengthening the Reporting of Observational Studies in Epidemiology (STROBE) reporting guideline.

Data from respondents to the Matriculating Student Questionnaire (MSQ) between academic years 2014 to 2015 and 2023 to 2024 were retrieved from the Association of American Medical Colleges. The MSQ asks students accepted to multiple medical schools the following 4-point Likert scale question, “In choosing the medical school you now (or will) attend, how important were:” (1) diversity of the student body and (2) the faculty? Responses of important and very important were used to indicate that students considered representational diversity important in medical school selection. Responses of not important and somewhat important were used to indicate that students considered representational diversity not important in medical school selection. Demographic data were self-reported and include sex, race, ethnicity, sexual orientation (heterosexual or lesbian, gay, bisexual [LGB]), and family income (≥$75 000 or <$75 000). Underrepresented in medicine (URiM) was defined as students identifying as American Indian or Alaska Native, Black, Hispanic, Native Hawaiian, or Pacific Islander.

Simple linear regression was used to determine annual percentage change (APC) in proportion of matriculants considering student and faculty diversity important. F-statistics determined differences in APC across groups. Analyses were performed using GraphPad Prism, with a 2-sided *P* < .05 significance threshold. Data were analyzed from March to April 2025.

## Results

Among 281 773 matriculants between 2014 to 2015 and 2023 to 2024, 189 176 (67.13%) responded to the MSQ. Among these, 99 714 (52.71%) identified as female, 46 537 (24.60%) Asian, 17 092 (9.03%) Black, 20 495 (10.83%) Hispanic, and 108 680 (57.45%) White. There were 17 045 LGB individuals (9.01%), and 55 692 individuals (29.44%) were from households with annual income less than $75 000. In 2014 to 2015, 43.89% and 33.09% of matriculants considered student and faculty diversity important, respectively, when selecting a medical school. By 2023 to 2024, these proportions increased to 62.52% and 54.87% (17.62–percentage point and 21.78–percentage point absolute increases for student and faculty diversity, respectively; *P* < .001). The highest proportions were observed in 2020, when 67.07% and 58.87% of matriculants considered student and faculty diversity important, respectively.

**Figure 1.**
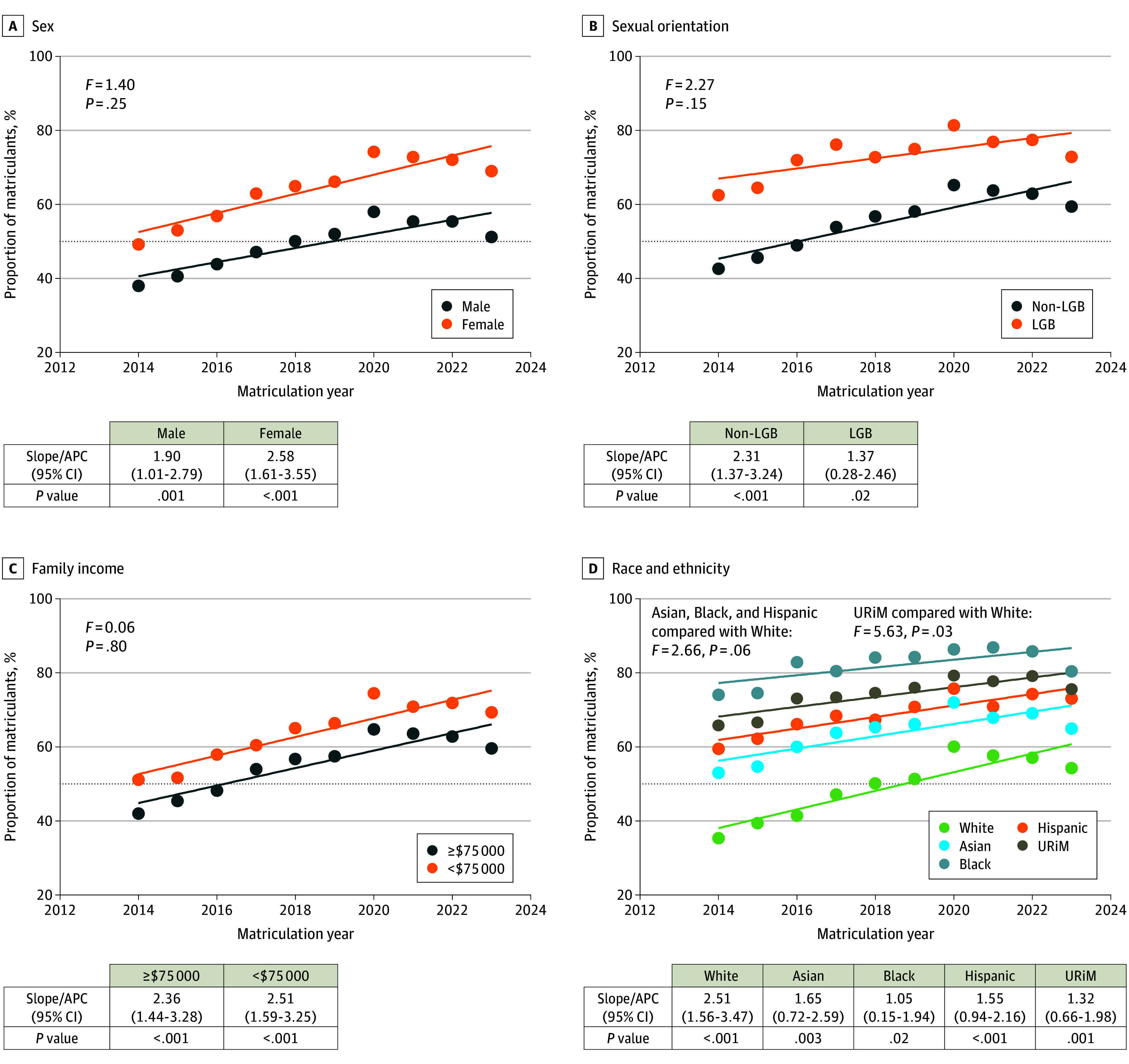
Proportion of Matriculants Considering Student Diversity Important Demographic data were self-reported on the American Medical College Application Service and Matriculating Student Questionnaire. Individuals with multiracial identities were included in each racial group they identified with (eg, individuals who identified as Asian and White were counted in both the Asian and White groups). Underrepresented in medicine (URiM) was defined as students identifying as Alaska Native, American Indian, Black, Hispanic, Native Hawaiian, or Pacific Islander. APC indicates annual percentage change; LGB lesbian, gay, and bisexual.

By 2023 to 2024, most matriculants across every demographic group rated student diversity as important, with a higher percentage of matriculants from historically marginalized backgrounds (URiM, LGB, and low-income) rating student diversity important when selecting medical schools (Figure 1). We observed higher APC in White compared with URiM matriculants (2.51% [95% CI, 1.56%-3.47%] vs 1.32% [95% CI, 0.66%-1.98%]; *P* = .03) and in heterosexual compared with LGB matriculants (2.31% [95% CI, 1.37%-3.24%] vs 1.37% [95% CI, 0.28%-2.46%]; *P* = .02). Male, female, lower-income, and higher-income matriculants had similar APCs (Figure 1).

**Figure 2.**
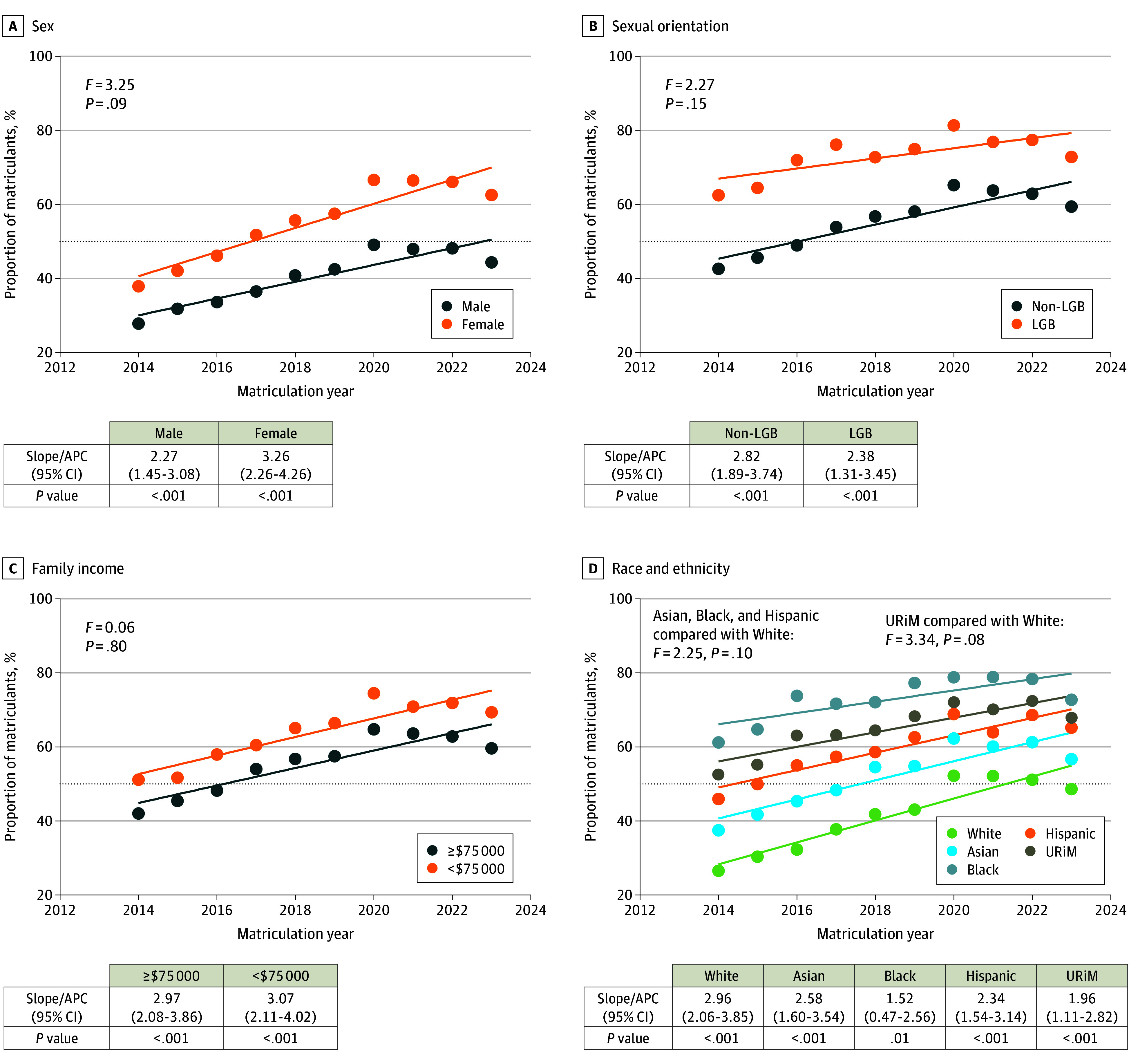
Proportion of Matriculants Considering Faculty Diversity Important Demographic data were self-reported on the American Medical College Application Service and Matriculating Student Questionnaire. Individuals with multiracial identities were included in each racial group they identified with (eg, individuals who identified as Asian and White were counted in both the Asian and White groups). Underrepresented in medicine (URiM) was defined as students identifying as Alaska Native, American Indian, Black, Hispanic, Native Hawaiian, or Pacific Islander. APC indicates annual percentage change; LGB lesbian, gay, and bisexual.

Between 2014 to 2015 and 2023 to 2024, a higher percentage of matriculants from historically marginalized backgrounds reported faculty diversity important when selecting medical schools (Figure 2). By 2023 to 2024, most matriculants across all demographic groups except White and male students rated faculty diversity important. The APCs of matriculants who considered faculty diversity important were not significantly different between female compared with male matriculants (3.26% [95% CI, 2.26%-4.26%] vs 2.27% [95% CI, 1.45%-3.08%]; *P* = .09) or for White compared with URiM matriculants (2.96% [95% CI, 2.06%-3.85%] vs 1.96% [95% CI, 1.11%-2.82%]; *P* = .08). Lower-income and higher-income matriculants, heterosexual and LGB students had similar increase in proportion of matriculants who considered faculty diversity important (Figure 2).

## Discussion

In this cohort study, we found a significant and consistent increase in the proportion of matriculants who considered student and faculty diversity important. This increase was observed across sex, income levels, sexual orientation, and racial and ethnic groups.

By the 2023 to 2024 academic year, most matriculants across all demographic groups felt that student and faculty diversity was important. There remains a potential disconnect between the mounting challenges to initiatives focused on diversity and diversity's critical role in improving health outcomes and the medical education learning environment.^6^

Our study has limitations. The response rate for the AAMC’s MSQ was 80.48%, which may limit the generalizability of findings. Nevertheless, to our knowledge, this is the largest study of medical school matriculants’ perspectives on the importance of diversity. Furthermore, students’ political beliefs may influence how they value diversity in medical school admissions. Support for diversity peaked in 2020, aligning with the COVID-19 pandemic and national response to the murder of George Floyd. Future research should examine how views shift amid rising anti–diversity, equity, and inclusion legislation.

## Notes

Supplement.
Data Sharing Statement
