# Supplementary material for: Student and Faculty Diversity in Medical School Selection
Source: JAMA Netw Open. 2025 Oct 21;8(10):e2533727. doi: 10.1001/jamanetworkopen.2025.33727 (PMC12541533; doi:10.1001/jamanetworkopen.2025.33727)
Supplement: Supplement. — Data Sharing Statement [file jamanetwopen-e2533727-s001.pdf]

Data Sharing Statement

Nguyen. Student and Faculty Diversity in Medical School Selection. *JAMA Netw Open*.
Published October 17, 2025. doi:10.1001/jamanetworkopen.2025.33727

Data
Data available: No
